# Ischemic Postconditioning Protects against Aged Myocardial Ischemia/Reperfusion Injury by Transcriptional and Epigenetic Regulation of miR-181a-2-3p

**DOI:** 10.1155/2022/9635674

**Published:** 2022-05-24

**Authors:** Guizhong Li, Ning Ding, Jiantuan Xiong, Shengchao Ma, Lin Xie, Lingbo Xu, Hui Zhang, Anning Yang, Yong Yang, Yideng Jiang, Huiping Zhang

**Affiliations:** ^1^School of Basic Medical Sciences, Ningxia Medical University, Yinchuan 750004, China; ^2^NHC Key Laboratory of Metabolic Cardiovascular Diseases Research, Ningxia Medical University, Yinchuan 750004, China; ^3^Ningxia Key Laboratory of Vascular Injury and Repair Research, Ningxia Medical University, Yinchuan 750004, China; ^4^Nuclear Medicine Center, People's Hospital of Ningxia Hui Autonomous Region, Yinchuan 750021, China; ^5^Department of Medical Genetics, Maternal and Child Health of Hunan Province, Changsha 410008, China

## Abstract

Ischemic postconditioning (IPostC) has been proposed as a strategy to mitigate the risk of ischemia/reperfusion (I/R) injury, and autophagy is involved in I/R-induced aged myocardial injury, while the underlying mechanism of IPostC-regulated autophagy is unknown. Here, we implemented miRNA sequencing analysis in aged cardiomyocytes to identify a novel miR-181a-2-3p after HPostC, which inhibits autophagy by targeting AMBRA1 in aged myocardium to protect I/R-induced aged myocardial injury. Mechanistically, we identified that IPostC can induce DNA hypomethylation and H3K14 hyperacetylation of miR-181a-2-3p promoter due to the decreased binding of DNMT3b and HDAC2 at its promoter, which contributes to enhancing the expression of miR-181a-2-3p. More importantly, cooperation of DNMT3b and HDAC2 inhibits the binding of c-Myc at the miR-181a-2-3p promoter in aged cardiomyocytes. In summary, IPostC attenuates I/R-induced aged myocardial injury through upregulating miR-181a-2-3p expression, which is an attribute to transcriptional and epigenetic regulation of its promoter. Our data indicate that miR-181a-2-3p may be a potential therapeutic target against I/R injury in aged myocardium.

## 1. Introduction

Ischemia-reperfusion (I/R) is a major pathological process that occurs in numerous organs and diseases [1]. Myocardial I/R injury is an unavoidable risk event for acute myocardial infarction, which leads to morbidity and mortality in elderly patients worldwide [2]. While significant advances have been made in the treatment of I/R injuries, cardioprotective therapeutics remains to be a significant challenge. Ischemic postconditioning (IPostC) is currently an effective approach to restrict the size of myocardial ischemia and reduce I/R injury [3]. However, the potential mechanism remains largely unknown; thus, it is important to explore new therapeutic targets to manage myocardial I/R injury.

Autophagy is an intracellular lysosomal degradative process that plays an important role in the homeostatic clearance of damaged cellular components via the degradation and recycling of cytosolic, long-lived proteins and organelles [4]. Previous studies have reported that autophagy participates in the pathological progress of I/R-injured heart, which has been proposed as a pivotal mechanism of cell death in myocardial I/R injury [5]. In the I/R-injured heart, excessive autophagy causes autophagic cell death, leading to subsequent damage to cells and tissues [6]. Accumulating evidences suggested that the inhibition of autophagic cell death protects cardiomyocytes during I/R injury [7]. However, there are also some studies indicating that enhanced lysosomal autophagy positively regulates myocardial I/R injury [8]. The beneficial and detrimental features of autophagy remain obscure, and more detailed investigations are needed to further clarify the role of autophagy during I/R-induced myocardial injury. Exploring and revealing the molecular mechanisms underlying the regulation of autophagy will provide a potential interventional strategy for treating an I/R-injured heart.

MicroRNA (miRNA) is an endogenous small noncoding RNA with 22-25 nucleotides in eukaryotes [9]. miRNA binds with the 3′-terminal nontranslation region (3′-UTR) of the target mRNA to degrade mRNA or inhibit its translation and thereby regulate the expression of the target gene to participate in various biological processes such as autophagy, apoptosis, and oxidative stress [10, 11]. Recent reports have provided evidence that many miRNAs are implicated in the development of ischemic cardiovascular disease. For instance, downregulation of miR-1, miR-let-7f, and miR-124 was observed in the cerebral cortex and hippocampus of mice following I/R [12–14]. Many studies have also shown that miRNA contributing to myocardial I/R injury, such as miR-204, miR-30e, and miR-221, was abnormally expressed in the myocardium and found to play a regulatory role in myocardial I/R injury by modulating autophagy [15]. Therefore, identification of I/R-associated miRNAs is important as it could provide new ideas for the treatment of myocardial I/R injury. Moreover, miRNA are also regulated by multiple factors. As epigenetic modulators, the dysregulation of the miRNA biogenesis pathway is mainly regulated at epigenetic, transcriptional, and posttranscriptional levels [16].

Aberrant miRNA expression is often associated with epigenetic regulation, such as DNA methylation and histone modifications [17]. Increasing evidence suggests that DNA methylation is a key epigenetic regulation of gene expression, including miRNA-coding genes [18]. Apart from stabilizing the inactive chromatin, DNA methylation could also inhibit the binding ability of transcription factors to gene promoters or recruit methyl-binding proteins that directly interact with transcription factors [19]. Unlike DNA methylation, histone acetylation usually opens the chromatin structure and increases chromatin accessibility and thereby allows transcription factors and the basal transcriptional machinery access to DNA easily. For example, HDAC6 and HDAC19 histone deacetylases might cooperate with AGL15 in silencing the complex that controls the abundance of miR-156 during embryogenic induction [20]. c-Myc is an oncogenic transcription factor that affects diverse cellular processes involved in cell growth, cell proliferation, and autophagy [21]. Recent studies have demonstrated that c-Myc plays a role in the transcriptional silencing of developmentally regulated genes via interaction with DNMT3b [22]. In addition, it was revealed that c-Myc recruited HAT that induced histone H4 acetylation in specific chromatin regions, thereby opening chromatin structures and amplifying the expression of target genes [23]. These findings suggest that gene expression is modulated by the coordinate effect of transcription factors and epigenetic modifications.

In this study, we found that miR-181a-2-3p involves in the protection of IPostC against I/R injury in aged myocardium by inhibiting autophagy via targeting AMBRA1. Mechanistically, downregulation of DNMT3b and HDAC2 by IPostC promotes the binding of c-Myc to the miR-181a-2-3p promoter, which in turn activates miR-181a-2-3p transcription. These results improve our understanding of cardioprotective mechanisms underlying IPostC against I/R injury in aged myocardium, which might contribute to the development of early diagnostic methods and novel therapy strategies for reperfusion injury of the ischemic cardiovascular disease.

## 2. Materials and Methods

### 2.1. Animals and Experimental Protocols

Male, aged Sprague-Dawley rats weighing between 600 g and 700 g (22-24 months) were purchased from Chengdu Da Shuo Biological Technology Co., Ltd (Chengdu, China). The aged rats were maintained in a standard condition (20-25°C, 50-60% humidity, with a 12 h light/12 h dark cycle) and maintained on a standard diet and water ad libitum. The aged rats were intraperitoneally injected with pentobarbital (50 mg/kg body weight), and the left anterior descending (LAD) coronary artery was ligated with a 5-0 silk suture (2 mm) below the left atrial appendage and the left edge of the pulmonary cone for 30 min to induce myocardial ischemia. After 30 min of ischemia, the silk thread was loosened for reperfusion for 180 min to establish the I/R model; IPostC was performed before the start of reperfusion, which consisted of 10 s of reperfusion and 10 s of ischemia for three cycles followed by 180 min reperfusion. Sham-operated rats underwent all surgical procedures except for the ligation step. During the surgical procedures, heart function was evaluated using a BL-420VHD biological functional demonstration system, which includes examining left ventricular systolic pressure (LVSP), left ventricular diastolic pressure (LVDP), and maximum rise/down velocity of left intraventricular pressure (±dp/dt_max_).

### 2.2. Cell Culture

Rat cardiomyocytes (H9C2 cells) were purchased from the Cell Bank of the Chinese Academy of Sciences (Shanghai, China) and cultured in Dulbecco's modified eagle's medium (DMEM; Gibco, USA) supplemented with 10% fetal bovine serum (FBS) and 100 U/mL streptomycin at 37°C in a humidified atmosphere containing 5% CO_2_. The aged cardiomyocyte model was established by induction with 8 mg/mL D-galactose (Sigma Aldrich, USA) for 9 days [24]. The environment of the anaerobic chamber was maintained as described previously [25]; then, the aged cardiomyocytes were subjected to hypoxia (95% N_2_, 5% CO_2_) for 180 min followed by 120 min of reoxygenation to establish the hypoxia/reoxygenation (H/R) model. After culture for 180 min under hypoxia, aged cardiomyocytes were cultured under three cycles of 5 min of hypoxia and 5 min of reoxygenation at the beginning of 120 min reoxygenation to establish the hypoxia postconditioning (HPostC) model. In addition, the aged cardiomyocytes treated with normoxia, H/R, and HPostC plus chloroquine (CQ, 100 *μ*M) treatment were harvested for follow-up experiments.

### 2.3. The Transfection of miR-181a-2-3p Mimic and Inhibitor in Aged Cardiomyocytes

miR-181a-2-3p mimic (5′-ACCACCAACCGUUGACUGU-3′), miR-181a-2-3p inhibitor (5′-ACAGUCAACGGUUGGUGGU-3′), and miRNA negative control (neg, 5′-UUGUACUACACAAAAGUACUG-3′) were obtained from Genepharma (Shanghai, China). The aged cardiomyocytes were transfected as previously described [26].

### 2.4. Knockdown of AMBRA1, DNMT3b, HDAC2, HDAC7, HDAC11, and c-Myc Expression by shRNA

The short hairpin (shRNA) sequences (Table S1) were cloned into ADV1 (U6/CMV-GFP) vectors and produced in HEK293 cells, which were purchased from Genepharma (Shanghai, China). The aged cardiomyocytes were transfected with shRNA according to the manufacturer's instruction.

### 2.5. Construction of Adenoviral Vector Expressing c-Myc

c-Myc cDNA was synthesized by Genepharma (Shanghai, China) and cloned into the pGLV5/EF-1aF/GFP-puro vector. The adenovirus vector (Ad) titer was determined by the median tissue culture infectious dose, and a titer of 1 × 10^9^ pfu/mL was estimated. The optimal multiplicity of infection (MOI) value was determined as 50. When the confluence of the aged cardiomyocytes was about 70%, the recombinant c-Myc adenovirus expression vector constructed by infection was used as the Ad-c-Myc group, and the aged cardiomyocytes were transfected with a negative control sequence as the Ad-GFP group. After 48 h transfection, aged cardiomyocytes were collected and used for follow-up experiments.

### 2.6. Detection of Lactic Dehydrogenase (LDH) and Cardiac Troponin I (cTnI) in Serum

Serum samples from the aged rat's abdominal aorta were collected after reperfusion. The release of LDH in serum was determined by LDH assay kits (Nanjing Jiancheng Bioengineering Institute, China). In brief, 50 *μ*L of 100 times diluted serum samples was added into a 96-well plate and mixed well with 50 *μ*L of the reaction mix. Sample absorbance was measured at 450 nm on a microplate reader and determined every 3 min for 60 min at 37°C in the dark. LDH activity of the samples was calculated according to the manufacturer's instructions. The levels of cTnI were measured with a two-site sandwich immunoassay kit (Nanjing Jiancheng Bioengineering Institute, China) in accordance with the manufacturer's instructions. First, 100 *μ*L standard or samples were added to each well for 2 h at 37°C. Then, the liquid was removed and added with 100 *μ*L biotin-antibody (1x) to incubate for 1 h at 37°C. Thereafter, the wells were washed 3 times before adding 100 *μ*L horseradish peroxidase-avidin (1x) to each well for 1 h at 37°C. After removing the liquid from each well and washing for 5 times, 90 *μ*L TMB substrate was added to each well for 15 min at 37°C in dark. At last, the reaction was stopped by adding 50 *μ*L stop solution to each well and read at 450 nm within 5 min.

### 2.7. Measurement of the Aged Myocardial Infarct Size

After 180 min reperfusion, the ligature around the LAD coronary artery was tightened, and then, 1 mL of 2% Evans blue dye (Sigma Aldrich, USA) in phosphate buffer saline (PBS) solution was injected into the carotid artery. Subsequently, the heart was immediately removed and frozen at -80°C, then cut into 6 sections with 2 mm thickness each and incubated in 2% 2,3,5-triphenyltetrazolium chloride (TTC) (Sigma Aldrich, USA) for 10 min at 37°C, and fixed in 4% formalin solution for 24 h and scanned. Normal areas were stained deep blue by Evans blue, ischemic areas at risk (AAR) were stained red by TTC, and infarct areas (IA) were white. The IA and AAR were analyzed using Image-Pro Plus 6.0. The infarct size was calculated as the percentage of IA over AAR (IA/AAR × 100%).

### 2.8. Transmission Electron Microscopy (TEM)

Heart tissues of 1 mm^3^ from the ischemic area at risk were fixed with 2.5% glutaraldehyde phosphate buffer (pH 7.4) and washed with 0.1 M phosphate buffer for 15 min for 3 times, 1% osmium, and 0.1 mol/L tetroxide phosphate-buffered saline, dehydrated with an acetone gradient, embedded in EPON-812 resin at 35°C overnight, and polymerized at 60°C for 48 h. Ultrathin sections were placed on a 200-mesh grid and stained with uranyl acetate and lead citrate followed by examination with transmission electron microscopy (Hitachi H-7650, Japan).

### 2.9. Analysis of Autophagic Flux

The aged cardiomyocytes were seeded in six-well plates and transfected with adenovirus harboring mRFP-GFP-LC3 (Hanbio Biotechnology, China). Autophagosomes (yellow puncta) and autolysosomes (red puncta) were detected by a confocal fluorescence microscope (Olympus Fluoview 1000, Japan). Autophagic flux was determined by evaluating the number of yellow and red puncta (puncta/cell were counted).

### 2.10. High-Throughput Sequencing of miRNAs

Total RNA was isolated from the aged cardiomyocytes using the TRIzol reagent (Invitrogen, USA). The small RNA sequencing libraries were generated using the NEBNext® Multiplex Small RNA Library Prep Set for Illumina® (NEB, USA) following the manufacturer's recommendations. Briefly, the NEB 3′ SR Adaptor was ligated to 3′ end of small RNAs. Afterward, the SR RT primer was hybridized to the excess of 3′ SR Adaptor and transformed the single-stranded DNA adaptor into a double-stranded DNA molecule. 5′ end adapter was ligated to 5′ ends of small RNAs. After first-strand cDNA synthesis and PCR amplification, the PCR products were purified and sequenced on an Illumina Hiseq 2500/2000 platform. After sequencing, the clean data (clean reads) were obtained by removing the low-quality reads from raw data. A certain range of length from clean reads was used for all the downstream analyses. The number of known miRNAs matures, and hairpins identified were 556 and 408, respectively. We used DESeq2 based on negative binomial distribution for miRNA differential expression analysis, and the input data was read count data obtained from miRNA expression level analysis. Subsequently, we performed Gene Ontology (GO) and KEGG enrichment analysis on the set of differentially expressed miRNA target genes for each group.

### 2.11. Quantitative Real-Time Polymerase Chain Reaction (qRT-PCR)

The first-strand cDNA was synthesized from total RNA using the Revert Aid first-strand cDNA synthesis kit (Thermo Scientific, USA). TB Green™ Premix Ex Taq™ (Takara Bio Inc., Japan) was used for amplification according to the manufacturer's instructions in a FTC3000P real-time PCR detection system (Funglyn, Canada). Glyceraldehyde-3-phosphate dehydrogenase (GAPDH) and U6 were used as a normalization control for mRNAs and miRNAs, respectively. The specific primers for miR-181a-2-3p are from the Bulge-Loop™ miRNA primer (RiboBio, China). The primer sequences of AMBRA1, HDACs, and c-Myc are listed in Table S2. The relative expression of mRNAs and miRNAs was analyzed using the 2^-*ΔΔ*ct^ method and normalized to the endogenous control.

### 2.12. Western Blot Analysis

Equal amounts of protein (30 *μ*g) were subjected to electrophoretic separation using sodium dodecyl sulfate-polyacrylamide gel electrophoresis (SDS-PAGE) gel and were subsequently transferred onto a polyvinylidene difluoride (PVDF) membrane (Millipore, USA). After blocking with nonfat milk in PBST for 2 h at room temperature, the PVDF membranes were incubated with primary antibodies at 4°C overnight. After washing with PBST, the membranes were incubated with secondary antibodies at room temperature for 1 h. The membranes were developed with an enhanced chemiluminescence solution (Millipore, USA) and analyzed using Image Lab 5.1 software.

### 2.13. Nested Methylation-Specific-Polymerase Chain Reaction (nMS-PCR)

Genomic DNA was purified using the Wizard® Genomic DNA Purification Kit (Promega, USA). An EZ DNA Methylation-Gold kit (Zymo Research, USA) was used to integrate DNA denaturation and bisulfite conversion processes. The miR-181a-2-3p primers used for the nMS-PCR assays are listed in Table S3. The procedure of nMS-PCR was performed as described previously [27]. The percentage of DNA methylation was calculated as methylation% = methylation/(methylation + unmethylation) × 100%.

### 2.14. MassARRAY Analysis

Genomic DNA from the aged cardiomyocytes was bisulfite-converted with the EpiTect Bisulfite Kit (QIAGEN, Germany) according to the manufacturer's instruction, followed by PCR designed to amplify the miR-181a-2-3p promoter. We designed primers for the miR-181a-2-3p promoter to cover the region with the most CpG sites. Our selected amplicon was in the promoter region of the gene. The mass spectra were collected using a MassARRAY Compact MALDI-TOF (BioMiao Biological Technology, China), and the spectra's methylation ratios were generated by the EpiTYPER software.

### 2.15. Luciferase Reporter Assay

miR-181a-2-3p promoter sequences (2117 bp) were obtained from the USCS Genome Browser. The transcription factor binding sites on the promoter of miR-181a-2-3p were analyzed by the JASPAR database. Fragments (-2000/+117, -1200/+117, -600/+117, and -200/+117) of the miR-181a-2-3p promoter were synthesized with the NheI/XhoI enzyme site at the ends and cloned into the firefly luciferase reporter vector pGL3-basic. Besides, the wild-type (WT) and mutant (Mut) AMBRA1 3′-UTR sequences containing the predicted binding site for miR-181a-2-3p luciferase reporter gene plasmids were generated by Genechem (Shanghai, China). After 48 h transfection, a luciferase reporter assay system (Promega, USA) was applied to monitor the relative luciferase activity.

### 2.16. Chromatin Immunoprecipitation (ChIP)

ChIP assays were performed for the aged cardiomyocytes using the EZ-ChIP™ Chromatin Immunoprecipitation Kit (Merck-Millipore, USA) according to the manufacturer's instructions. Immunoprecipitation was performed with indicated antibody and normal mouse IgG (negative control) at 4°C overnight. 1/100 of total aged cardiomyocyte lysate was used as an internal control (input). PCR analyzed the precipitated DNA using specific primers (the primer sequences are listed in Table S4). The amplified product was then examined by electrophoresis, and the signals were calculated as the percentage of input.

### 2.17. Coimmunoprecipitation (Co-IP) Assay

The aged cardiomyocytes were lysed in NP40 lysis buffer containing protease inhibitor on ice. After centrifugation, the supernatant was incubated with indicated antibody or normal rabbit IgG at 4°C overnight followed by incubation with protein A/G plus-agarose (Santa Cruz Technology, USA). The immunoprecipitates were washed three times in lysis buffer and boiled in SDS sample buffer for 5 min. Then, the immune complex was separated by SDS-PAGE and proceeded for Western blot analysis.

### 2.18. Statistical Analysis

Data are expressed as the mean ± SD from at least three independent experiments. The results were analyzed with GraphPad Prism 6.0 software. One-way ANOVA, Student-Newman-Keul's test (comparisons between multiple groups), or unpaired Student's *t* test (between two groups) was used as appropriate. A value of *P* < 0.05 was considered statistically significant.

### 2.19. Study Approval

All the treatment and experimental protocols for rats were approved by the Committee on the Ethics of Animal Experiments of Ningxia Medical University, and the ethics approval number is 2019-067. The animal procedures were performed in strict accordance with the Animal Research: Reporting of In Vivo Experiments guidelines.

## 3. Results

### 3.1. IPostC Alleviates I/R Injury via Autophagy in Aged Myocardium

**Figure 1:**
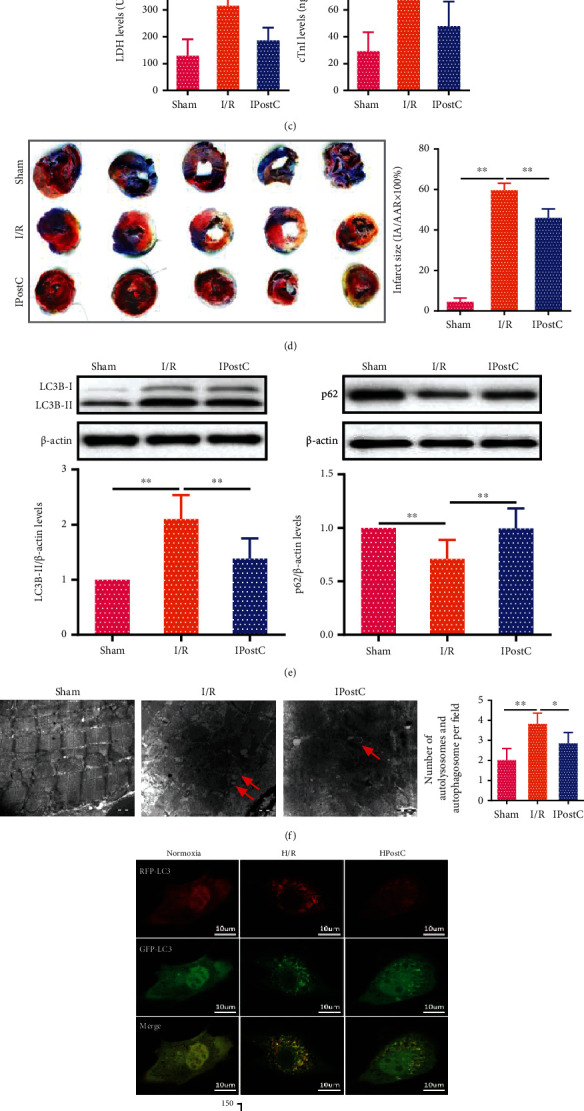
IPostC attenuates I/R injury of aged myocardium via autophagy. (a) Pattern map of the treatment of the aged myocardium and aged cardiomyocytes. (b) Aged cardiac function was demonstrated in terms of left ventricular systolic pressure (LVSP), left ventricular diastolic pressure (LVDP), and maximum rise/down velocity of left intraventricular pressure (±dp/dT_max_) by BL-420 system after IPostC (*n* = 6). (c) The serum levels of cardiac marker enzymes lactate dehydrogenase (LDH) and cardiac troponin-I (cTnI) were detected by ELISA in aged myocardium after 3 h of reperfusion (*n* = 6). (d) Representative heart sections stained with Evans blue and 2,3,5-triphenyltetrazolium chloride (TTC) of the infarct size in aged rats after IPostC. Normal areas were stained by Evans's blue; TTC staining (red) indicated the areas at risk (AAR); white color indicated the infarct areas (IA) (*n* = 6). (e) Western blot analysis of LC3B-II and p62 expression in aged myocardium after IPostC (*n* = 6). (f) Transmission electron microscopy (TEM) images showed the autolysosomes and autophagosomes in aged myocardium after IPostC (scale bar = 1000 nm). Red arrows indicated the autolysosomes and autophagosomes (*n* = 6). The number of autolysosomes and autophagosomes per field was accounted for in the histogram. (g) Fluorescence of mRFP-GFP-LC3 in aged cardiomyocytes after hypoxia postconditioning (HPostC) was detected by confocal microscopy. Yellow puncta represented the merged fluorescence of RFP and GFP fluorescence which indicated autophagosomes, whereas free red puncta (RFP only) represented autolysosomes (scale bar = 10 *μ*m) (*n* = 3). (h) Western blot analysis of LC3B-II and p62 expression in aged cardiomyocytes after HPostC in the absence and presence of chloroquine (CQ) (*n* = 3). Data were presented as mean ± SD. ^∗^*P* < 0.05,  ^∗∗^*P* < 0.01.

Ischemic postconditioning (IPostC) is defined as an effective strategy to reduce myocardial ischemia-reperfusion (I/R) injury. Firstly, the aged rats were subjected to sham, ischemia-reperfusion (I/R), and ischemia postconditioning (IPostC) to establish an animal model; then, the aged cardiomyocytes were induced with 8 mg/mL D-galactose for 9 days to establish the normoxia, hypoxia/reoxygenation (H/R), and hypoxia postconditioning (HPostC) cell model (Figure 1(a)). We subsequently verified the protective effects of IPostC on myocardial I/R injury. As shown in Figure 1(b), IPostC significantly improved the decrease of left ventricular systolic pressure (LVSP), left ventricular diastolic pressure (LVDP), and maximum rise/down velocity of left intraventricular pressure (±dp/dT_max_) induced by I/R injury. The serum lactate dehydrogenase (LDH) and cardiac troponin I (cTnI) levels were also reduced after IPostC (Figure 1(c)). Remarkably, Evans blue and TTC staining results indicated that IPostC decreased I/R-induced infarct size in isolated hearts of the aged rats (Figure 1(d)), which demonstrated the cardioprotective effects of IPostC on myocardial I/R injury.

To investigate whether IPostC attenuates I/R injury via autophagy in aged myocardium, the LC3B-II and p62 expression of aged myocardium were measured by Western blot after IPostC. The results showed that I/R increased the LC3B-II expression, which was reversed by IPostC, while I/R decreased the p62 expression that could be recovered by IPostC (Figure 1(e)). Transmission electron microscopy (TEM) also showed that the number of autophagosomes or autolysosomes was decreased after IPostC (Figure 1(f)). Given that autophagy is a gradual and dynamic process, mRFP-GFP-LC3 adenovirus infection was applied to monitor the effect of HPostC on the autophagy flux in aged cardiomyocytes. It was found that both the autophagosomes and autolysosomes were remarkably increased in aged cardiomyocytes exposed to H/R, while HPostC decreased the number of autophagosomes and autolysosomes (Figure 1(g)), implying that autophagy participates in the protective effect of HPostC on aged cardiomyocytes. Thus, we further investigated the effect of HPostC on autophagy through an inhibitor of autophagy, chloroquine (CQ). The results showed that CQ increased the LC3B-II expression in aged cardiomyocytes, while the expression of p62 was downregulated (Figure 1(h)). Collectively, these results suggest that IPostC have a protective effect on aged myocardial I/R injury via autophagy.

### 3.2. miR-181a-2-3p Attenuates Aged Myocardial I/R Injury by Targeting AMBRA1 in IPostC

**Figure 2:**
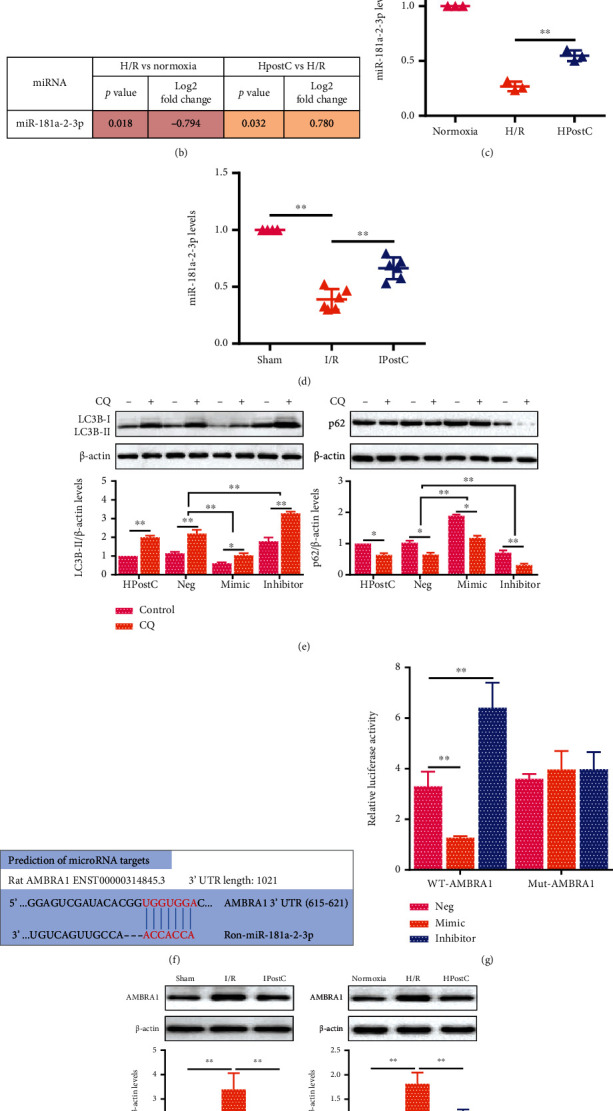
IPostC inhibits aged myocardial I/R injury through miR-181a-2-3p. (a) The cluster heat map showed the differentially expressed miRNAs in aged cardiomyocytes after HPostC (fold change ≥ 2, *P* ≤ 0.05). Red and blue indicate the miRNA expression level. The original expression values of miRNAs were normalized using *Z*-score normalization. (b) The detailed schematic of miR-181a-2-3p from miRNA sequencing analysis. (c, d) The expression of miR-181a-2-3p was validated by qRT-PCR both in aged cardiomyocytes (*n* = 3) after HPostC and in aged myocardium (*n* = 6) after IPostC. (e) Western blot analysis of LC3B-II and p62 expression in aged cardiomyocytes transfected with miR-181a-2-3p mimic or inhibitor after HPostC in the presence of CQ (*n* = 3). (f) Predicted binding of miR-181a-2-3p to AMBRA1 3′-UTR by TargetScan (http://www.targetscan.org/vert_72/). (g) The reporter constructs containing the wild type (WT) and mutant type (Mut) 3′-UTR regions of AMBRA1 were transfected into HEK293 cells with miR-181a-2-3p mimic or inhibitor. Relative luciferase activities were normalized by Renilla luciferase activities (*n* = 6). (h) AMBRA1 expression was detected by Western blot both in aged myocardium after IPostC (left, *n* = 6) and aged cardiomyocytes after HPostC (right, *n* = 3). (i) Western blot analysis of AMBRA1 expression in aged cardiomyocytes transfected with miR-181a-2-3p mimic or inhibitor after HPostC (*n* = 3). (j) The expression of LC3B-II and p62 was detected by Western blot in aged cardiomyocytes transfected with sh-AMBRA1 in the presence of CQ (*n* = 3). (k) Fluorescent GFP-LC3 and RFP-LC3 puncta in aged cardiomyocytes transfected with sh-AMBRA1 after HPostC. Numbers of autophagosomes and autolysosomes in each cell were quantified (scale bar = 10 *μ*m) (*n* = 3). Data were presented as the mean ± SD. ^∗^*P* < 0.05,  ^∗∗^*P* < 0.01.

To screen the potential miRNAs involved in autophagy of aged cardiomyocytes, we performed miRNA sequencing analysis in aged cardiomyocytes after HPostC. Hierarchical clustering of miRNA expression showed 62 differentially expressed miRNAs (32 upregulated and 30 downregulated) in H/R compared with normoxia and 14 differentially expressed miRNAs in HPostC (2 upregulated and 12 downregulated) (Figure 2(a)). KEGG analysis of 14570 genes revealed 13111 enriched pathways, and 16 pathways were found to be responsible for autophagy in H/R compared with normoxia (Figure S1a). Meanwhile, 15 pathways were found to be responsible for autophagy after HPostC (Figure S1b). Among them, miR-181a-2-3p is the only miRNA expressed simultaneously in aged cardiomyocytes (Figure 2(b)). The qRT-PCR analysis showed that the expression level of miR-181a-2-3p was downregulated in aged cardiomyocytes after H/R, while HPostC partially inhibited the decrease of miR-181a-2-3p induced by H/R (Figure 2(c), Figure S1c). Similar results were also observed in aged myocardium after IPostC (Figure 2(d)). To study the effect of miR-181a-2-3p on autophagy, we transfected miR-181a-2-3p mimic or inhibitor into aged cardiomyocytes (Figure S1d). As shown in Figure 2(e), CQ-induced LC3B-II accumulation was significantly decreased by miR-181a-2-3p mimic in aged cardiomyocytes after HPostC, while the effect of CQ on LC3B-II level was elevated in aged cardiomyocytes with miR-181a-2-3p inhibitor, implying that HPostC suppressed autophagy via miR-181a-2-3p in aged cardiomyocytes.

Next, to identify the molecular mechanism of miR-181a-2-3p involved in autophagy of aged cardiomyocytes, miR-181a-2-3p and its potential target gene were predicted by TargetScan. Among these genes, we selected autophagy-related target genes to further confirm by the luciferase reporter assay (Figure S1e). miRNA-targeted analysis further found that both rat and human miR-181a-2-3p had binding sites with AMBRA1 (Figure 2(f) and Figure S1f, g). We utilized a pGL3-AMBRA1-3′-UTR luciferase reporter vector to check if the sequence was in response to miR-181a-2-3p. Cotransfection of miR-181a-2-3p mimic with AMBRA1-3′-UTR reporter (WT-AMBRA) in HEK293 cells diminished the relative luciferase activity, and cotransfection of the miR-181a-2-3p inhibitor with WT-AMBRA promoted the relative luciferase activity, while the reporter gene vector containing mutation predicting sequences of AMBRA1 (Mut-AMBRA1) had no impact on miR-181a-2-3p mimic and inhibitor (Figure 2(g)). These results suggested that miR-181a-2-3p could directly target AMBRA1 by binding to the putative sequences within its 3′-UTR. Meanwhile, we found that the expression of AMBRA1 in aged cardiomyocytes was significantly increased after H/R compared with normoxia but reversed after HPostC. Consistent results were observed in aged myocardium after IPostC (Figure 2(h)). Accordingly, Western blot analysis showed that miR-181a-2-3p inhibited AMBRA1 expression in aged cardiomyocytes after HPostC (Figure 2(i)). In addition, AMBRA1 downregulation inhibited CQ-induced LC3B-II accumulation and reduced GFP-LC3 puncta, while the expression of p62 was significantly increased in aged cardiomyocytes transfected with sh-AMBRA1 after HPostC (Figures 2(j) and 2(k)). These data indicate that miR-181a-2-3p ameliorates I/R injury by inhibiting autophagy in aged myocardium through AMBRA1.

### 3.3. IPostC Restrains DNA Methylation of miR-181a-2-3p Promoter by DNMT3b in Aged Myocardium

**Figure 3:**
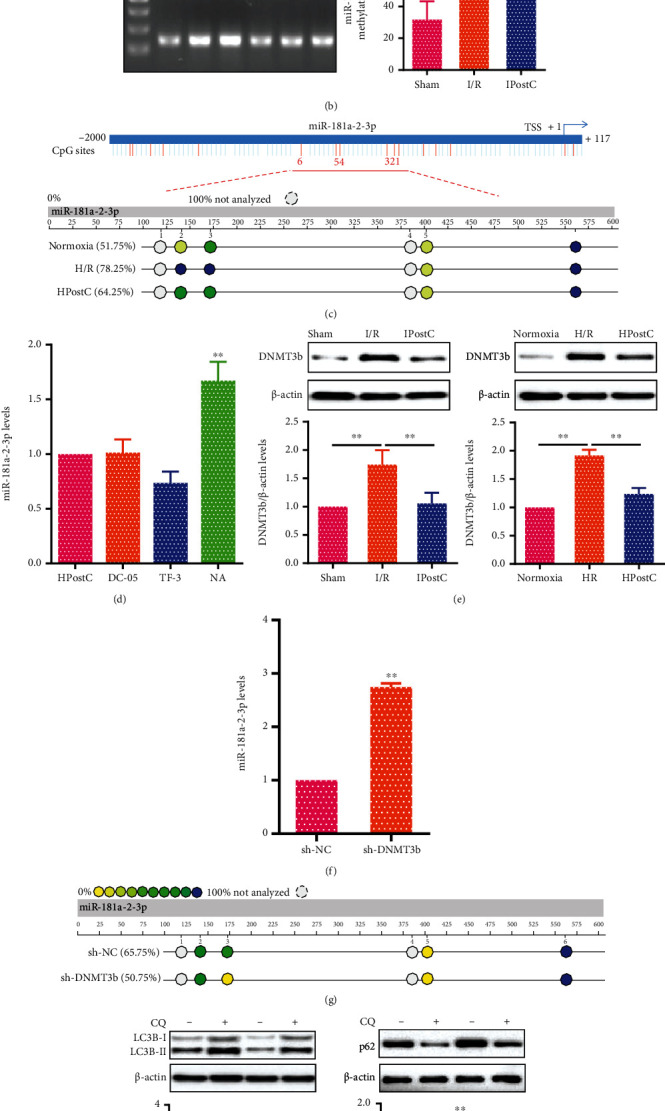
IPostC inhibits autophagy by DNA hypomethylation of miR-181a-2-3p in aged myocardium. (a) The promoter activity of miR-181a-2-3p was evaluated by the luciferase reporter assay. Different deletion fragments of miR-181a-2-3p promoter (-2000/+117, -1200/+117, -600/+117, and -200/+117) cotransfected into HEK293 cells with Renilla luciferase vector (internal control), and the results were represented as firefly luciferase activity normalized to Renilla luciferase activity (*n* = 3). (b) DNA methylation levels of miR-181a-2-3p promoter were detected by nMS-PCR in aged myocardium after IPostC (*n* = 6). The reactions for unmethylated and methylated DNA are denoted by U and M, respectively. (c) MassARRAY analysis was performed to determine the methylation levels of miR-181a-2-3p promoter from -2000 to +117 in aged cardiomyocytes after HPostC. Each circle represents a single CpG analyzed. Methylation frequencies are displayed in a color code that extends from yellow (lower methylation frequencies) to blue (higher methylation frequencies). White circles indicate not analyzed methylation values due to CpGs with high or low mass Dalton peaks falling outside the conservative window of reliable detection for the EpiTYPER software. (d) miR-181a-2-3p expression was validated by qRT-PCR in aged cardiomyocytes treated with DC_05 (DNMT1 inhibitor), Theaflavin-3 (TF-3, DNMT3a inhibitor), and Nanaomycin A (NA, DNMT3b inhibitor) after HPostC, respectively (*n* = 3). (e) DNMT3b expression was detected by Western blot both in aged myocardium after IPostC (*n* = 6) and in aged cardiomyocytes after HPostC (*n* = 3). (f) The expression of miR-181a-2-3p was analyzed by qRT-PCR in aged cardiomyocytes transfected with sh-DNMT3b after HPostC (*n* = 3). (g) MassARRAY analysis was used to measure the methylation levels of miR-181a-2-3p promoter in aged cardiomyocytes transfected with sh-DNMT3b after HPostC. (h) Western blot analysis of LC3B-II and p62 expression in aged cardiomyocytes transfected with sh-DNMT3b in the presence of CQ (*n* = 3). Data were presented as the mean ± SD. ^∗^*P* < 0.05,  ^∗∗^*P* < 0.01.

To elucidate the underlying mechanism of miR-181a-2-3p in aged myocardium after IPostC. The sequence of miR-181a-2-3p promoter was analyzed by UCSC Genome Browser, and a rich GC% was enriched in the promoter of miR-181a-2-3p (Figure S2). Next, several fragments of miR-181a-2-3p promoter (-2000/+117, -1200/+117, -600/+117, and -200/+117) were inserted into the firefly luciferase vector pGL3-basic, and the luciferase reporter assay revealed that the region from -1200 to -600 had the highest promoter activity (Figure 3(a)), indicating a possible regulatory element of miR-181a-2-3p transcription in the region. Subsequently, DNA methylation of the miR-181a-2-3p promoter region was examined in aged myocardium. As shown in Figure 3(b), the promoter of miR-181a-2-3p was hypermethylated in I/R injury, which was reversed by IPostC. In addition, MassARRAY analysis was used to evaluate DNA methylation levels of miR-181a-2-3p promoter in aged cardiomyocytes after HPostC; the results showed that HPostC significantly decreased DNA methylation of miR-181a-2-3p promoter (Figure 3(c)).

Generally, DNA methyltransferases (DNMTs) are responsible for the regulation of DNA methylation at CpG islands [28]. To better illustrate whether DNMTs (DNMT1, DNMT3a, and DNMT3b) are involved in DNA methylation of miR-181a-2-3p promoter after HPostC, the aged cardiomyocytes were treated with DC_05 (DNMT1 inhibitor), Theaflavin-3 (TF-3, DNMT3a inhibitor), and Nanaomycin A (NA, DNMT3b inhibitor) after HPostC. We found that NA enhanced the expression of miR-181a-2-3p, while there were no significant changes in aged cardiomyocytes treated with DC_05 or TF-3 (Figure 3(d)). Furthermore, we observed that the expression of DNMT3b was decreased both in aged myocardium after IPostC and in aged cardiomyocytes after HPostC, indicating DNMT3b contributed to DNA methylation of miR-181a-2-3p promoter in IPostC (Figure 3(e)). Then, DNMT3b was suppressed in aged cardiomyocytes by sh-DNMT3b for follow-up experiments (Figure S3). It was found that the expression of miR-181a-2-3p was significantly increased, while its methylation level was reduced in aged cardiomyocytes transfected with sh-DNMT3b after HPostC (Figures 3(f) and 3(g)). Moreover, the LC3B-II expression was decreased, while p62 expression was increased in aged cardiomyocytes transfected with sh-DNMT3b after HPostC, in which CQ increased the LC3B-II expression and decreased p62 expression (Figure 3(h)). These results indicate that IPostC enhances the miR-181a-2-3p expression through DNA methylation in aged myocardium.

### 3.4. HDAC2 Regulates miR-181a-2-3p Expression via H3K14 Acetylation in Aged Myocardium in IPostC

**Figure 4:**
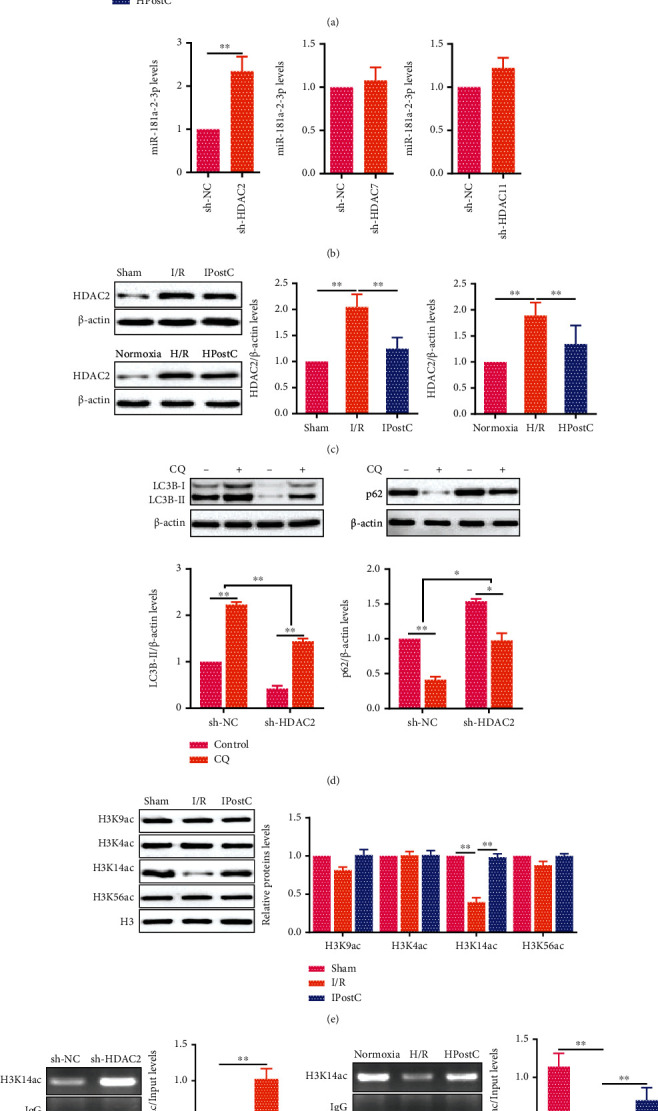
IPostC reduces autophagy via H3K14ac enrichment at miR-181a-2-3p promoter in aged myocardium. (a) The expression of HDACs was detected by qRT-PCR in aged cardiomyocytes after HPostC (*n* = 3). (b) The miR-181a-2-3p expression was detected by qRT-PCR in aged cardiomyocytes transfected with sh-HDAC2, sh-HDAC7, or sh-HDAC11 (*n* = 3). (c) Western blot analysis of HDAC2 expression both in aged myocardium after IPostC (*n* = 6) and in aged cardiomyocytes after HPostC (*n* = 3). (d) LC3B-II and p62 expression was detected by Western blot after the aged cardiomyocytes were transfected with sh-HDAC2 in the presence of CQ (*n* = 3). (e) Western blot analysis of H3K9ac, H3K4ac, H3K14ac, and H3K56ac in aged myocardium after IPostC (*n* = 6). Histone 3 (H3) was used as a loading control. (f) The enrichment of H3K14ac at miR-181a-2-3p promoter was identified by ChIP in aged cardiomyocytes after HPostC (*n* = 3). DNA from each ChIP sample was normalized by the corresponding input sample. (g) The enrichment of H3K14ac at miR-181a-2-3p promoter was detected by ChIP in aged cardiomyocytes transfected with sh-HDAC2 after HPostC (*n* = 3). Data were presented as mean ± SD. ^∗^*P* < 0.05,  ^∗∗^*P* < 0.01.

Considering that HDACs (enzyme that regulates histone deacetylation) are involved in gene expression via histone deacetylation, to inspect the relationship between miR-181a-2-3p and histone deacetylation, the expression of HDACs was analyzed by qRT-PCR in aged cardiomyocytes. It was found that HDAC2, HDAC7, and HDAC11 had obvious changes in aged cardiomyocytes after H/R or HPostC (Figure 4(a)). We further transfected sh-HDAC2, sh-HDAC7, and sh-HDAC11 into aged cardiomyocytes (Figure S4). The results showed that the expression of miR-181a-2-3p was increased after the aged cardiomyocytes were transfected with sh-HDAC2, while HDAC7 and HDAC11 had no effect on miR-181a-2-3p expression (Figure 4(b)). Western blot analysis also confirmed that the expression of HDAC2 was decreased both in aged myocardium after IPostC and in aged cardiomyocytes after HPostC (Figure 4(c)). To investigate the effects of HDAC2 on HPostC-reduced autophagy of aged cardiomyocytes, Western blot analysis showed that the expression of LC3B-II was decreased and the expression of p62 was increased after the aged cardiomyocytes were transfected with sh-HDAC2 (Figure 4(d)), implying that HDAC2 promoted autophagy in aged cardiomyocytes after HPostC.

Next, the bioinformatics analysis was performed to analyze the Gene Ontology-biological process of HDAC2. As shown in Figure S5, HDAC2 was associated with “histone H3 deacetylation” (red underline), specifically “histone deacetylase activity (H3-K14)” (red underline, GO ID: 0031078). The reliability of this prediction was confirmed by Western blot analysis, which showed that the level of H3K14ac was increased in aged myocardium after IPostC (Figure 4(e)). Consistent with the Western blot result, ChIP showed that there was a significant enrichment of H3K14ac at miR-181a-2-3p promoter in aged cardiomyocytes (Figure 4(f)). Meanwhile, the enrichment of H3K14ac at miR-181a-2-3p promoter was increased in aged cardiomyocytes transfected with sh-HDAC2 after HPostC (Figure 4(g)). Taken together, these results demonstrate that HDAC2 inhibits H3K14ac to reduce miR-181a-2-3p expression in aged cardiomyocytes.

### 3.5. DNMT3b and HDAC2 Cooperatively Regulate miR-181a-2-3p Expression of Aged Cardiomyocytes

**Figure 5:**
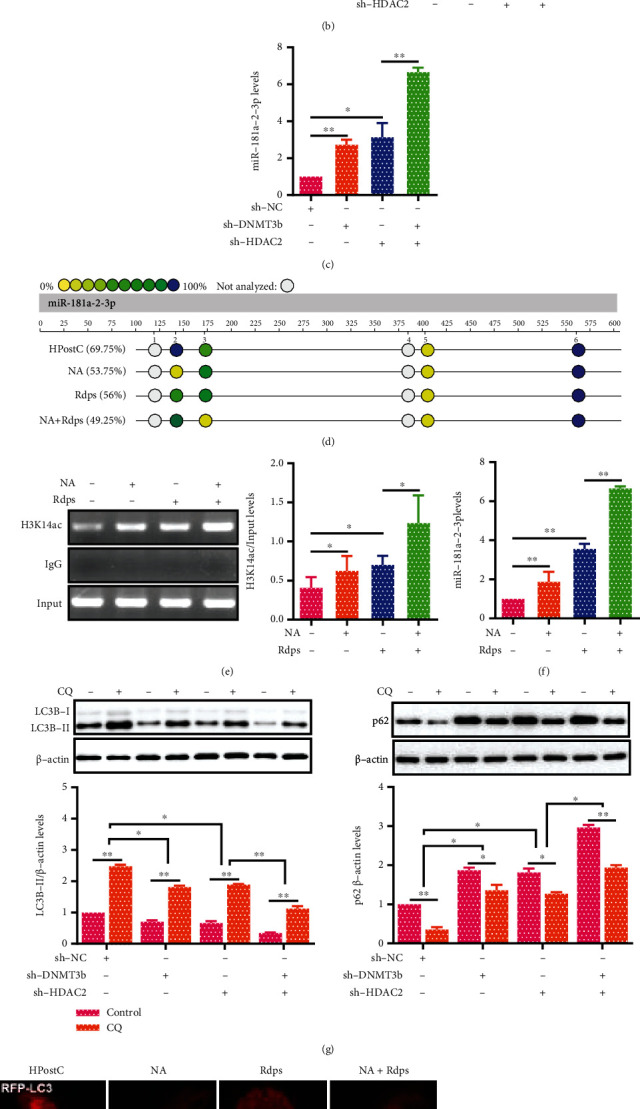
Cooperation of DNMT3b and HDAC2 involved in the regulation of miR-181a-2-3p and autophagy in aged cardiomyocytes. After the aged cardiomyocytes were transfected with sh-DNMT3b and sh-HDAC2 in HPostC, then (a) MassARRAY analysis detected the DNA methylation level of miR-181a-2-3p promoter. (b) ChIP measured enrichment of H3K14ac at miR-181a-2-3p promoter (*n* = 3). (c) qRT-PCR was performed to measure miR-181a-2-3p expression (*n* = 3). (d–f) The levels of DNA methylation and H3K14ac at miR-181a-2-3p promoter and the expression of miR-181a-2-3p were detected after the aged cardiomyocytes were treated with NA (DNMT3b inhibitor) and romidepsin (Rdps, HDAC2 inhibitor) (*n* = 3). (g) Western blot analysis of LC3B-II and p62 expression in aged cardiomyocytes transfected with sh-DNMT3b and/or sh-HDAC2 in the presence of CQ (*n* = 3). (h) Representative confocal fluorescent images of mRFP-GFP-LC3 after aged cardiomyocytes were treated with NA and Rdps (scale bar = 10 *μ*m). Numbers of autophagosomes and autolysosomes in each cell were quantified (*n* = 3). Data were presented as the mean ± SD. ^∗^*P* < 0.05,  ^∗∗^*P* < 0.01.

According to the above results, DNMT3b and HDAC2 were key enzymes involved in DNA methylation and H3K14ac at miR-181a-2-3p promoter, respectively. To elucidate the potential relationship between DNMT3b and HDAC2 in regulating miR-181a-2-3p expression, sh-DNMT3b and sh-HDAC2 were transfected into aged cardiomyocytes. As shown in Figures 5(a) and 5(b), the DNA methylation level of miR-181a-2-3p promoter was reduced, and the enrichment of H3K14ac at miR-181a-2-3p promoter was increased in aged cardiomyocytes transfected with sh-HDAC2 or sh-DNMT3b, and a further enhancement was detected when using them collectively. Consistently, the expression of miR-181a-2-3p was significantly increased in aged cardiomyocytes transfected with sh-DNMT3b and/or sh-HDAC2 after HPostC (Figure 5(c)). The aged cardiomyocytes were treated with NA and Rdps to further assess the impact of DNMT3b and HDAC2 on the expression of miR-181a-2-3p. As expected, the DNA methylation level of miR-181a-2-3p promoter was decreased, while H3K14ac enrichment at miR-181a-2-3p promoter and miR-181a-2-3p expression were obviously increased (Figures 5(d)–5(f)). These results indicate that HDAC2 and DNMT3b synergistically inhibit the expression of miR-181a-2-3p in aged cardiomyocytes after HPostC. To investigate whether DNMT3b and HDAC2 are cooperatively involved in autophagy of aged cardiomyocytes, we detected the expression of LC3B-II and p62 by Western blot. As shown in Figure 5(g), the expression of LC3B-II was apparently decreased in aged cardiomyocytes with sh-HDAC2 or sh-DNMT3b alone, and a further decrease was observed when using them jointly, which was accompanied by the opposite alteration of p62 expression. Consistent with the Western blot results, a marked decline in the number of both autophagosomes and autolysosomes was observed in the combination treatment of NA and Rdps (Figure 5(h)). In summary, these data suggest that DNMT3b and HDAC2 cooperatively regulate miR-181a-2-3p expression to affect autophagy in aged cardiomyocytes.

### 3.6. c-Myc Binds and Activates miR-181a-2-3p Transcription in Aged Myocardium after IPostC

**Figure 6:**
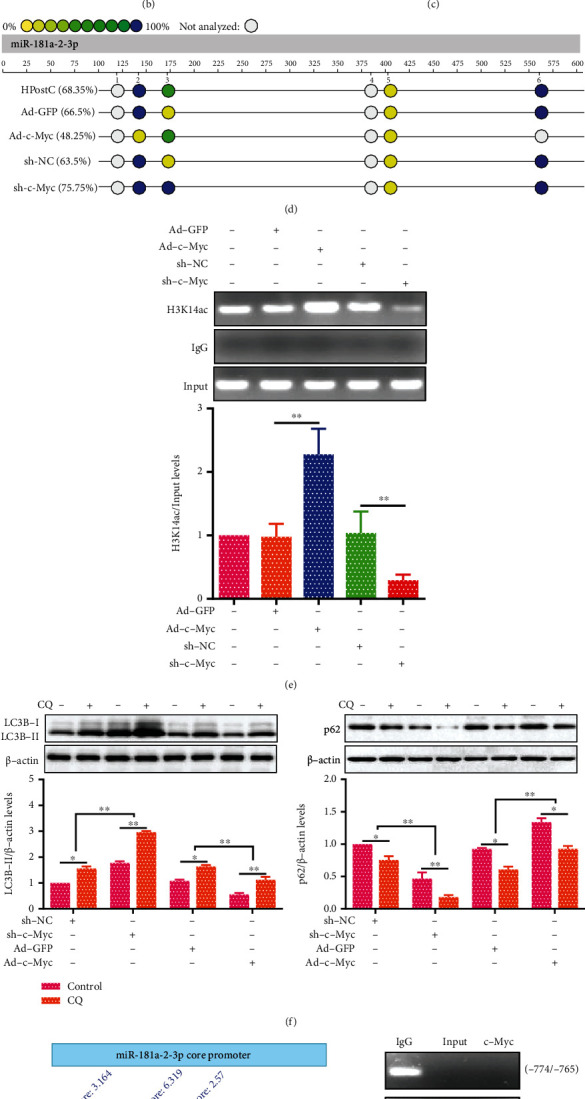
c-Myc enhances miR-181a-2-3p expression via directly binding to its promoter. (a) qRT-PCR and Western blot analysis of c-Myc expression both in aged myocardium after IPostC (*n* = 6) and in aged cardiomyocytes after HPostC (*n* = 3). (b) The activity of miR-181a-2-3p promoter was detected by luciferase reporter assay in HEK293 cells transfected with Ad-c-Myc or sh-c-Myc (*n* = 3). After the aged cardiomyocytes were transfected with Ad-c-Myc or sh-c-Myc, then (c) qRT-PCR analysis of the expression of miR-181a-2-3p (*n* = 3), (d) MassARRAY analysis of DNA methylation level of miR-181a-2-3p promoter (*n* = 3), and (e) ChIP analysis of the enrichment of H3K14ac at miR-181a-2-3p prompter (*n* = 3). (f) Western blot analysis of LC3B-II and p62 expression after the aged cardiomyocytes were transfected with sh-c-Myc or Ad-c-Myc in the presence of CQ (*n* = 3). (g) Schematic diagram showed the locations of predicted c-Myc-binding sites (black hollow circle) in the core promoter region (-1200/-600) of miR-181a-2-3p. c-Myc binding at miR-181a-2-3p promoter in aged cardiomyocytes was assessed by ChIP using c-Myc antibody. (h) The promoter activity of miR-181a-2-3p with wild type (WT) or the mutant (Mut) binding sites of c-Myc (Mut1: -1126/-1117, Mut2: -806/-797) was detected by luciferase reporter assay in HEK293 cells (*n* = 3). Data were presented as the mean ± SD. ^∗^*P* < 0.05,  ^∗∗^*P* < 0.01.

c-Myc, a transcription factor critical for regulating cellular functions, has been reported to participate in the regulation of gene transcription [29]. To investigate whether c-Myc could regulate miR-181a-2-3p transcription in aged myocardium, we first detected c-Myc expression in aged myocardium by qRT-PCR and Western blot, respectively. The results showed that c-Myc was significantly increased in aged myocardium after IPostC. Similar results were obtained in aged cardiomyocytes after HPostC (Figure 6(a)). Next, we transfected Ad-c-Myc and sh-c-Myc into aged cardiomyocytes (Figure S6), and the luciferase reporter assay and qRT-PCR analysis were performed to detect the activity of miR-181a-2-3p promoter and its expression. As shown in Figures 6(b) and 6(c), miR-181a-2-3p promoter activity and its expression were increased in aged cardiomyocytes transfected with Ad-c-Myc, while transfection with sh-c-Myc obtained opposite results, suggesting that c-Myc may be involved in the transcriptional regulation of miR-181a-2-3p.

Given that transcriptional activation of miR-181a-2-3p is dependent on DNA methylation and H3K14ac of its promoter, we further investigated the effect of c-Myc on DNA methylation and H3K14ac at miR-181a-2-3p promoter. The results showed that the DNA methylation level of miR-181a-2-3p promoter was decreased in aged cardiomyocytes transfected with Ad-c-Myc, while the enrichment of H3K14ac at miR-181a-2-3p promoter was increased. However, transfection with sh-c-Myc markedly reversed these effects (Figures 6(d) and 6(e)). In addition, CQ-induced LC3B-II accumulation was significantly decreased in aged cardiomyocytes transfected with Ad-c-Myc, which can be reversed by sh-c-Myc transfection, and the expression of p62 showed a contrary manner (Figure 6(f)). To assess the binding of c-Myc to miR-181a-2-3p promoter, we first analyzed miR-181a-2-3p promoter sequence using the JASPAR database (https://ngdc.cncb.ac.cn/databasecommons/database/id/176) and found three putative c-Myc binding sites at miR-181a-2-3p basic core promoter: -1126/-1117, -806/-797, and -774/-765 (Figure S7). The ChIP assay further showed that there was strong binding of c-Myc with miR-181a-2-3p promoter at -806/-797 and -1126/-1117 sites (Figure 6(g)). Meanwhile, the luciferase reporter assay also indicated that both the -806/-797 and -1126/-1117 sites are essential for the binding of c-Myc to miR-181a-2-3p promoter (Figure 6(h)). Taken together, these results indicate that c-Myc transcriptionally activates miR-181a-2-3p by directly binding to miR-181a-2-3p promoter in aged myocardium.

### 3.7. HDAC2 Interacts with DNMT3b to Suppress c-Myc Binding at miR-181a-2-3p Promoter in Aged Myocardium

**Figure 7:**
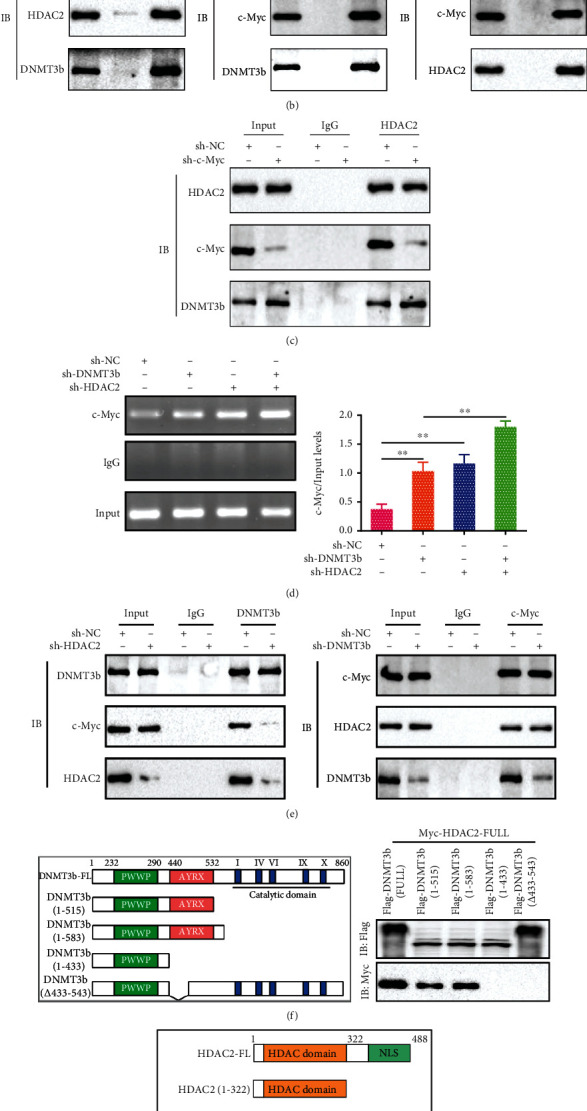
Cooperation of DNMT3b and HDAC2 inhibits the binding of c-Myc to miR-181a-2-3p promoter in aged cardiomyocytes after HPostC. (a) The binding of HDAC2, c-Myc, and DNMT3b to the miR-181a-2-3p promoter was analyzed by ChIP in aged cardiomyocytes. (b) The interaction between c-Myc, HDAC2, and DNMT3b was detected by coimmunoprecipitation (Co-IP) in aged cardiomyocytes. (c) Co-IP was used to detect the interaction between HDAC2 and DNMT3b or c-Myc in aged cardiomyocytes transfected with sh-c-Myc. (d) The binding of c-Myc to the miR-181a-2-3p promoter was analyzed by ChIP after the aged cardiomyocytes were transfected with sh-HDAC2 and sh-DNMT3b, respectively (*n* = 3). (e) The combination of HDAC2 or DNMT3b with c-Myc was detected by Co-IP in aged cardiomyocytes transfected with sh-HDAC2 and sh-DNMT3b, respectively. (f) Mapping the interface of HDAC2 with DNMT3b by Co-IP assay after HEK293 cells were cotransfected with plasmids encoding different Flag-tagged DNMT3b fragments (DNMT3b-FL, 1-515, 1-583, 1-433, *Δ*433-543) and Myc-tagged HDAC2. (g) HEK293 cells were cotransfected with plasmids encoding different Myc-tagged HDAC2 fragments (HDAC2-FL, 1-332, 323-488) and Flag-tagged DNMT3b (1-515). Data were presented as the mean ± SD. ^∗∗^*P* < 0.01.

To investigate whether c-Myc regulating miR-181a-2-3p transcription is cooperated with HDAC2 and DNMT3b in aged cardiomyocytes after HPostC, the ChIP assay was performed to detect the binding of c-Myc, HDAC2, and DNMT3b to miR-181a-2-3p promoter. As shown in Figure 7(a), c-Myc, HDAC2, and DNMT3b were intensely bound to miR-181a-2-3p promoter. The reciprocal Co-IP assay also showed that c-Myc, HDAC2, and DNMT3b robustly interacted with each other, suggesting mutual interaction of the above three proteins as a ternary complex (Figure 7(b)). We next determined the mode of the c-Myc-HDAC2-DNMT3b interaction. The Co-IP assay showed that the interaction between HDAC2 and DNMT3b was not affected in aged cardiomyocytes transfected with sh-c-Myc (Figure 7(c)). Meanwhile, the binding of c-Myc at miR-181a-2-3p promoter was markedly enhanced in aged cardiomyocytes transfected with sh-DNMT3b and sh-HDAC2 (Figure 7(d)). These results indicated that cooperative DNMT3b binding with HDAC2 is integral to the mechanism by which c-Myc regulates transcription of the miR-181a-2-3p. Moreover, we also observed that the c-Myc binding with DNMT3b was dramatically reduced in aged cardiomyocytes transfected with sh-HDAC2 (Figure 7(e), left), whereas the interaction of c-Myc with HDAC2 was not affected after transfecting with sh-DNMT3b (Figure 7(e), right), meaning DNMT3b and c-Myc are independent of HDAC2 to form a complex for the regulation of miR-181a-2-3p transcription.

To further clarify the exact regions of DNMT3b participating in the interaction with HDAC2, we constructed a series of truncated constructs of HDAC2 and DNMT3b. The plasmids encoding different Flag-tagged DNMT3b fragments (DNMT3b-FL, 1-515, 1-583, 1-433, *Δ*433-543) were coexpressed together with plasmids encoding Myc-tagged HDAC2 in HEK293 cells, and the Co-IP assay was performed with anti-Myc or anti-Flag. Obviously, the results showed that the N-terminal region of DNMT3b that harbors the AYRX domain interacts with HDAC2 (Figure 7(f)). Conversely, we found that the strong binding ability of the N-terminal of HDAC2 was required for the interaction with DNMT3b (1-515) (Figure 7(g)). The above observation demonstrates that the 1-515 domain of DNMT3b binds to the 1-332 domain of HDAC2. Taken together, these findings indicate that c-Myc, HDAC2, and DNMT3b can form a ternary complex and that c-Myc enhances miR-181a-2-3p expression in cooperation with HDAC2 and DNMT3b.

## 4. Discussion

Myocardial infarction mediated by I/R injury can induce irreversible myocardial cell loss and myocardial dysfunction, which is the main cause of morbidity and mortality in elderly patients [30]. IPostC is a cardioprotective phenomenon, which has been indicated to be associated with better preserved cardiac function and improved clinical outcomes [31]. Previous studies also showed the benefit of IPostC in the animal model, such as limited infarct size, preserved left ventricular function, inhibited necrosis and apoptosis, reduced myocardial edema, and coronary microembolization [32]. In this study, we demonstrated that IPostC significantly improves cardiac function in aged myocardium. More importantly, IPostC showed significant benefit in aged patients who suffered from acute myocardial infarction, which is consistent with our findings in an aged rat model.

Autophagy is a dynamic process of phagocytosis and degradation of autologous cytoplasmic proteins or organelles and is involved in the pathology of myocardial I/R injury. It has been reported that autophagy is prevalent in the acute and chronic ischemic heart, and the role of autophagy in the cardiac tissue depends on the degree of induction and duration of the injury [33]. Hamacher­Brady et al. reported that I/R-induced autophagy of myocardial HL-1 cells significantly decreased, suggesting its cardioprotective role in myocardial I/R injury response [34]. On contrary, excessive autophagy during reperfusion increased cell death, which is harmful to the heart [35]. Consistent with Aoyagi's report, our study found that the autophagy in aged myocardium significantly decreased after IPostC, suggesting that modulation of the autophagic pathway provides protection over a prolonged period of time. Therefore, inhibition of autophagy may be a potential therapeutic option for the treatment or prevention of myocardial I/R injury.

It is well known that miRNAs are aberrantly expressed in cardiovascular diseases; it is able to serve as circulating biomarkers and connect with the pathophysiology of many cardiovascular diseases [36]. Evidence indicates that miRNAs have been widely involved in I/R injury [37]. However, limited studies have investigated alterations in miRNA expression levels in aged myocardium treated with IPostC following I/R injury. miR-181a-2-3p, a member of the miR-181 family, is located at the intron of the Nr6a1 gene. It has been reported that miR-181a expression was significantly altered by I/R compared with time-matched nonischemic controls, while postconditioning significantly inhibited these alterations [38]. Here, we found that the expression of miR-181a-2-3p was significantly downregulated in I/R-injured aging myocardium, whereas IPostC treatment promoted its expression. These findings demonstrate that I/R-induced expression changes of miR-181a-2-3p are specifically counterregulated by subsequent postconditioning stimuli. Thus, we hypothesize that IPostC exerts its cardioprotective effect by upregulation of miR-181a-2-3p. Recently, it was found that cardiac-specific overexpression of miR-181a could be of therapeutic interest to target Aldo-MR pathway-induced pathological cardiac remodeling [39]. Consistent with these studies, the present study revealed that overexpression of miR-181a-2-3p inhibited autophagy in aged cardiomyocytes, suggesting miR-181a-2-3p as a novel autophagy regulator to promote cardioprotective effects of IPostC against I/R injury. miRNAs are known to regulate cardiovascular diseases via interaction with 3′-UTR of their target genes leading to mRNA degradation and posttranslational repression [40]. Here, we identified AMBRA1 as a target gene of miR-181a-2-3p; the inhibition of AMBRA1 results in a decreased autophagic activity. Thus, miR-181a-2-3p limits uncontrolled or potentially harmful autophagic activity via suppressing AMBRA1 expression. More importantly, numerous steps involved in the biogenesis of miRNAs leave lots of scope for spatiotemporal regulation of miRNA accumulation at the posttranscriptional level. Our data highlight the complexity of this regulation and show that miRNAs and their action on targets are regulated at multiple levels.

Given the cooperative linkage between DNMTs and HDACs in regulating gene expression, several in vivo studies have provided clues on the functional connection between these two epigenetic modifications [41]. A recent report indicated that promoter methylation is closely associated with miRNA expression [18]. Besides, HDAC1/2 inhibitor (romidepsin) decreased NO-induced IRF1 and PUMA expression, which attenuates cell death and tissue injury in I/R injury [42]. DNMT1 inhibitor 5-Aza-CdR enhanced the protective effect for NCOA4-siRNA of cell injury during diabetes myocardial I/R injury [43]. This study revealed that the downregulation of HDAC2 and DNMT3b by IPostC results in increased H3K14ac levels and decreased DNA methylation of the miR-181a-2-3p promoter thus promoting miR-181a-2-3p expression, implying that IPostC-induced miR-181a-2-3p expression is epigenetically regulated by DNA hypomethylation and histone acetylation in aged myocardium. Cho et al. found that silenced ribosomal RNA genes could be activated by the methyltransferase inhibitor or histone deacetylase inhibitors, suggesting that DNA hypomethylation and histone acetylation collaboratively activate gene expression, which further supports our conclusions [44].

The c-Myc transcription factor is a well-known nuclear oncoprotein that has bounded to several molecules to regulate the transcription of genes involved in cell growth and apoptosis [45, 46]. Here, our study suggests that c-Myc enhances autophagy in aged cardiomyocytes, and miR-181a-2-3p is transcriptionally regulated by c-Myc. However, transcription factors are not the only layer of regulation controlling gene function. We further demonstrate that the collaboration of DNA hypomethylation with H3K14ac promotes c-Myc binding to miR-181a-2-3p promoter, leading to the activation of miR-181a-2-3p transcription. It illustrates that transcription factor binding patterns at miRNA promoters are affected by DNA methylation and histone acetylation.

## 5. Conclusions

**Figure 8:**
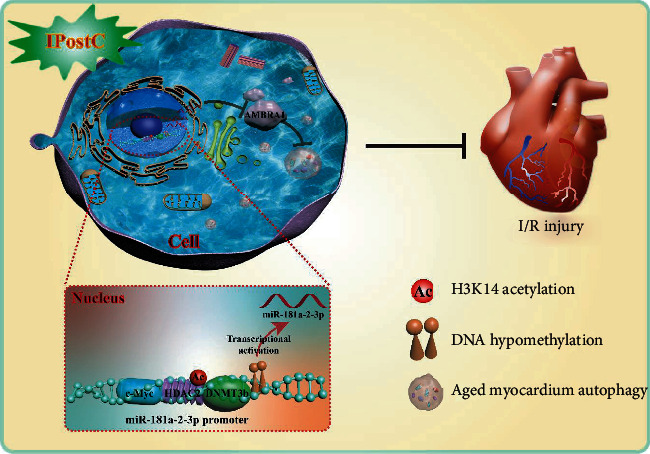
Ischemic postconditioning protects against aged myocardial ischemia/reperfusion injury by transcriptional and epigenetic regulation of miR-181a-2-3p. IPostC attenuates I/R-induced aged myocardial injury through upregulating miR-181a-2-3p expression to inhibit autophagy via targeting AMBRA1. Furthermore, the interaction of DNA hypomethylation with H3K14ac promotes the binding of c-Myc at miR-181a-2-3p promoter, leading to the activation of miR-181a-2-3p transcription under IPostC thereby protecting the aged myocardium from I/R injury.

In summary, our results suggest that cooperation of DNA hypomethylation with H3K14ac promotes the binding of c-Myc at miR-181a-2-3p promoter, leading to the activation of miR-181a-2-3p transcription in aged myocardium after IPostC. miR-181a-2-3p further protects the aged myocardium from I/R injury via targeting AMBRA1 to inhibit autophagy. A deep understanding of the protection mechanism by which IPostC is against I/R injury in aged myocardium will be beneficial for the potential clinical application perspectives of miR-181a-2-3p as a helpful biomarker or therapeutic target in ischemic myocardial infarction (Figure 8).

## Data Availability

The datasets used and/or analyzed during the current study are available from the corresponding authors on reasonable request.

## Supplementary Materials

Supplementary Materials Figure S1: miR-181a-2-3p is a key molecule inhibiting autophagy of aged cardiomyocytes in HPostC. Figure S2: the CpG island of miR-181a-2-3p was identified by UCSC Genome Browser and Meth Primer. Figure S3: the protein expression of DNMT3b was detected by Western blot in aged cardiomyocytes transfected with sh-DNMT3b (sh-DNMT3b-1, sh-DNMT3b-2). Figure S4: the expression of HDAC2, HDAC7, and HDAC11 in aged cardiomyocytes. Figure S5: the GO biological process analysis of HDAC2. Figure S6: c-Myc expression in aged cardiomyocytes. Figure S7: c-Myc binding sites at miR-181a-2-3p promoter. Table S1: sequences of shRNAs against AMBRA1, DNMT3b, HDAC2, HDAC7, HDAC11, and c-Myc. Table S2: primer sequences for qRT-PCR. Table S3: primer sequences for nMS-PCR. Table S4: primer sequences for ChIP assay.
