# Supplementary material for: Ischemic Postconditioning Protects against Aged Myocardial Ischemia/Reperfusion Injury by Transcriptional and Epigenetic Regulation of miR-181a-2-3p
Source: Oxid Med Cell Longev. 2022 May 24;2022:9635674. doi: 10.1155/2022/9635674 (PMC9155916; doi:10.1155/2022/9635674)
Supplement: Supplementary Materials — Figure S1: miR-181a-2-3p is a key molecule inhibiting autophagy of aged cardiomyocytes in HPostC. Figure S2: the CpG island of miR-181a-2-3p was identified by UCSC Genome Browser and Meth Primer. Figure S3: the protein expression of DNMT3b was detected by Western blot in aged cardiomyocytes transfected with sh-DNMT3b (sh-DNMT3b-1, sh-DNMT3b-2). Figure S4: the expression of HDAC2, HDAC7, and HDAC11 in aged cardiomyocytes. Figure S5: the GO biological process analysis of HDAC2. Figure S6: c-Myc expression in aged cardiomyocytes. Figure S7: c-Myc binding sites at miR-181a-2-3p promoter. Table S1: sequences of shRNAs against AMBRA1, DNMT3b, HDAC2, HDAC7, HDAC11, and c-Myc. Table S2: primer sequences for qRT-PCR. Table S3: primer sequences for nMS-PCR. Table S4: primer sequences for ChIP assay. [file 9635674.f1.docx]

**Supplementary Figures**


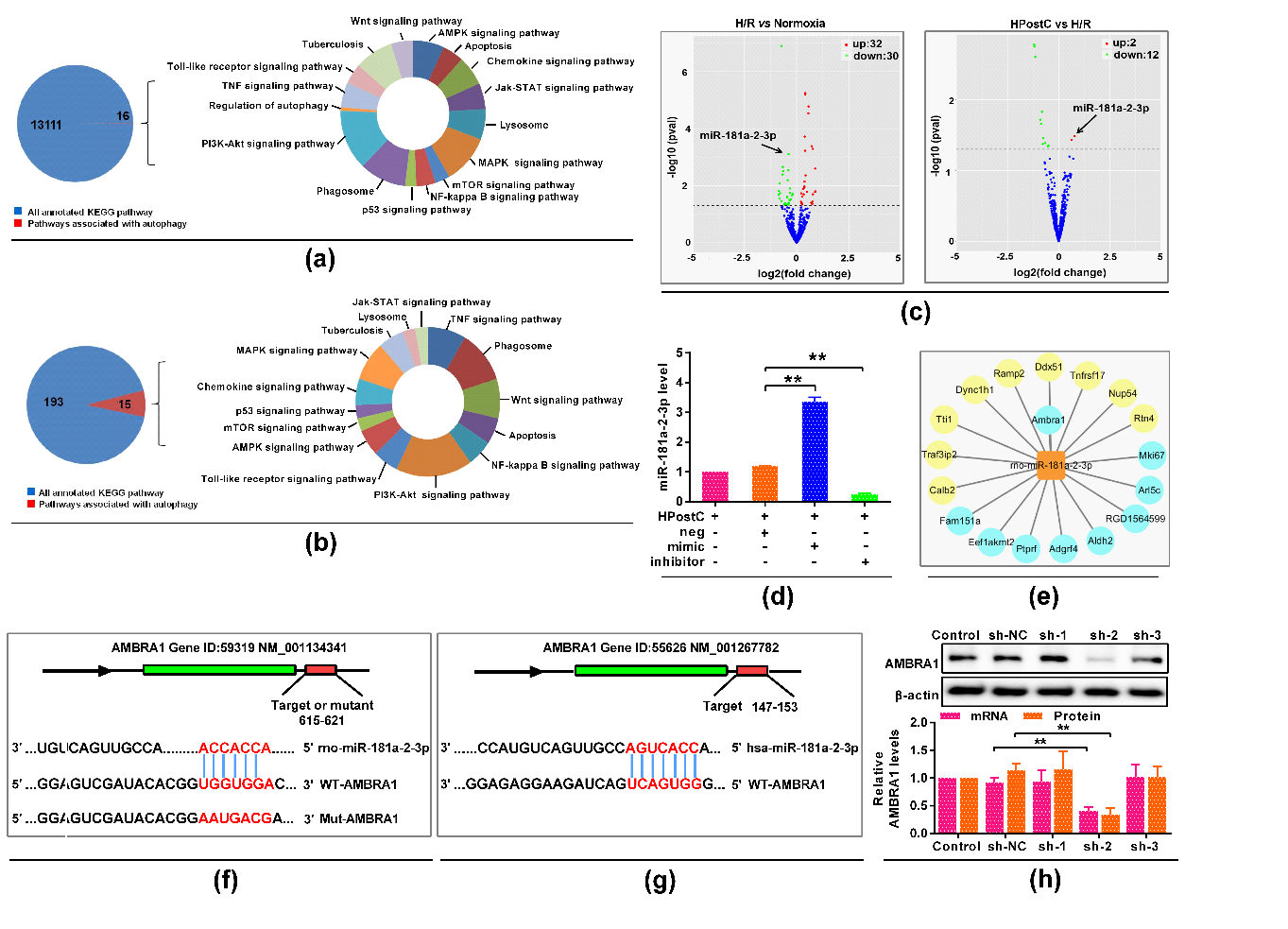


**Figure S1. miR-181a-2-3p is a key molecule inhibiting autophagy of aged cardiomyocytes in HPostC. (a)** 13111 pathways were annotated, including 16 pathways related to autophagy in H/R compared with Normoxia (Left). The exact pathways and genes were provided (Right). **(b)** 193 pathways were annotated, including 15 pathways related to regulation of autophagy after HPostC (Left). The exact pathways and genes were provided (Right). **(c)** Expression profiles of miRNAs in aged cardiomyocytes after HR or HPostC. The volcano plot was constructed using fold-change values and *P*-values. The red and green dots in the plot represent significantly upregulated and downregulated miRNAs, while the blue dots represent miRNAs with no significant difference. **(d)** qRT-PCR was performed to detect miR-181a-2-3p expression in aged cardiomyocytes transfected with miR-181a-2-3p mimic or inhibitor (n=3). **(e)** miRNA-gene network for miR-181a-2-3p. The network was built using targeted genes and predicted interactions from the TargetScan databases. Orange node represents miR-181a-2-3p, yellow and blue nodes represent target genes; their relationship is represented by the edges. **(f)** The binding site for rno-miR-181a-2-3p within wild-type (WT) 3'-UTR of AMBRA1 and its mutant (Mut) version by site mutagenesis. **(g)** The binding site for hsa-miR-181a-2-3p within wild-type (WT) 3'-UTR of AMBRA1. **(h)** qRT-PCR and Western blot analysis of AMBRA1 expression in aged cardiomyocytes transfected with sh-AMBRA1 (sh-AMBRA1-1, sh-AMBRA1-2, sh-AMBRA1-3) (n=3). Data were presented as mean ± SD. ***P*<0.01.

**
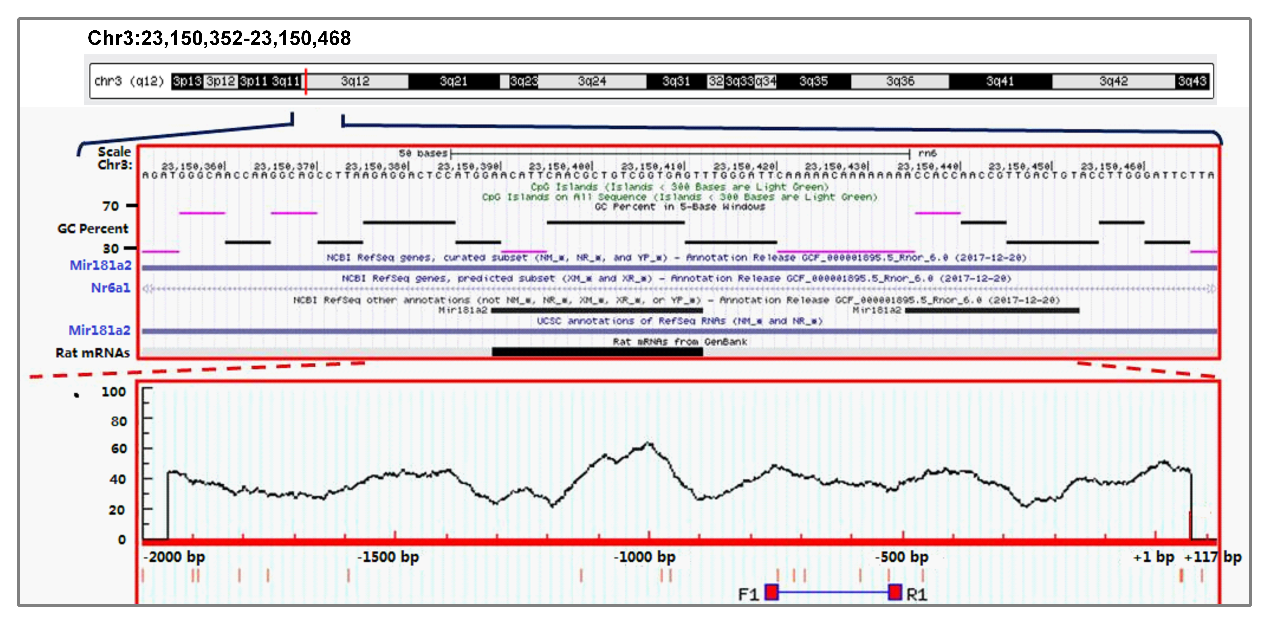
**

**Figure S2. The CpG island of miR-181a-2-3p was identified by UCSC Genome Browser and Meth Primer.** Middle panel presents the CpG Island, relative % GC content and mRNA was obtained from UCSC (http://genome.ucsc.edu). Lower panel: Meth Primer database (http://www.urogene.org/cgi-bin/Methprimer2/Meth Primer.cgi) analysis of the CpG sites of miR-181a-2-3p promoter ranging from -2000 to +117 bp.


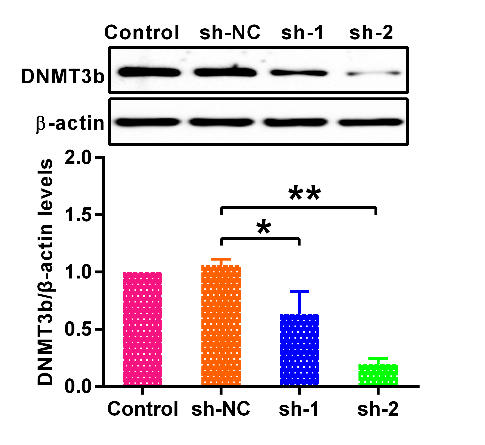


**Figure S3.** The protein expression of DNMT3b was detected by Western blot in aged cardiomyocytes transfected with sh-DNMT3b (sh-DNMT3b-1, sh-DNMT3b-2) (n=3). Data were presented as mean ± SD. **P*<0.05, ***P*<0.01.


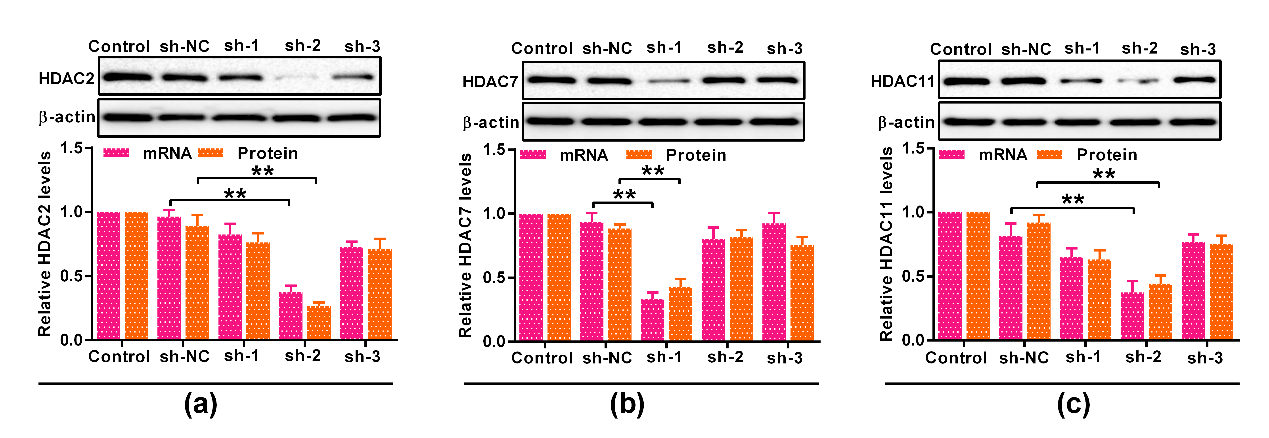


**Figure S4. The expression of HDAC2, HDAC7 and HDAC11 in aged cardiomyocytes. (a-c)** HDAC2, HDAC7 and HDAC11 were detected by qRT-PCR and Western blot after the aged cardiomyocytes were transfected with sh-HDAC2 (sh-HDAC2-1, sh-HDAC2-2, sh-HDAC2-3), sh-HDAC7 (sh-HDAC7-1, sh-HDAC7-2, sh-HDAC7-3), or sh-HDAC11 (sh-HDAC11-1, sh-HDAC11-2, sh-HDAC11-3), respectively (n=3). Data were presented as mean ± SD. ***P*<0.01.


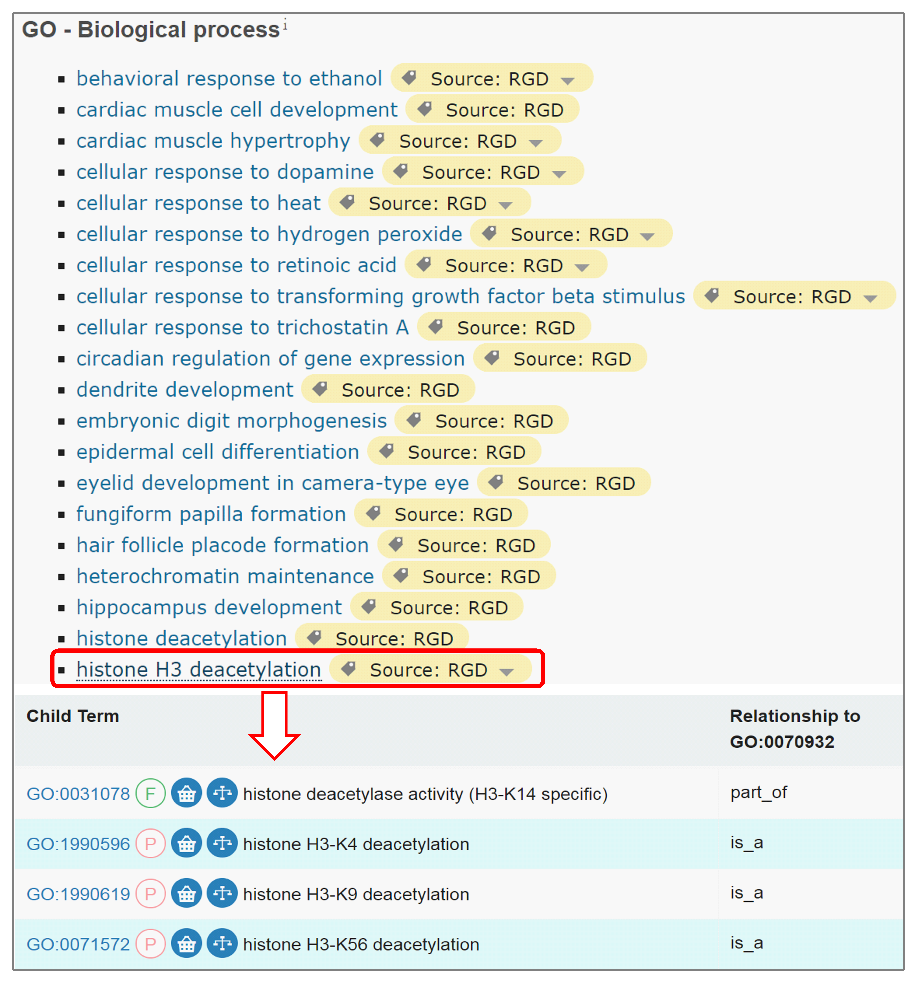


**Figure S5. The GO biological process analysis of HDAC2.** Gene Ontology (GO) analysis of biological process of HDAC2 associated with histone H3 deacetylation, specifically H3K14.


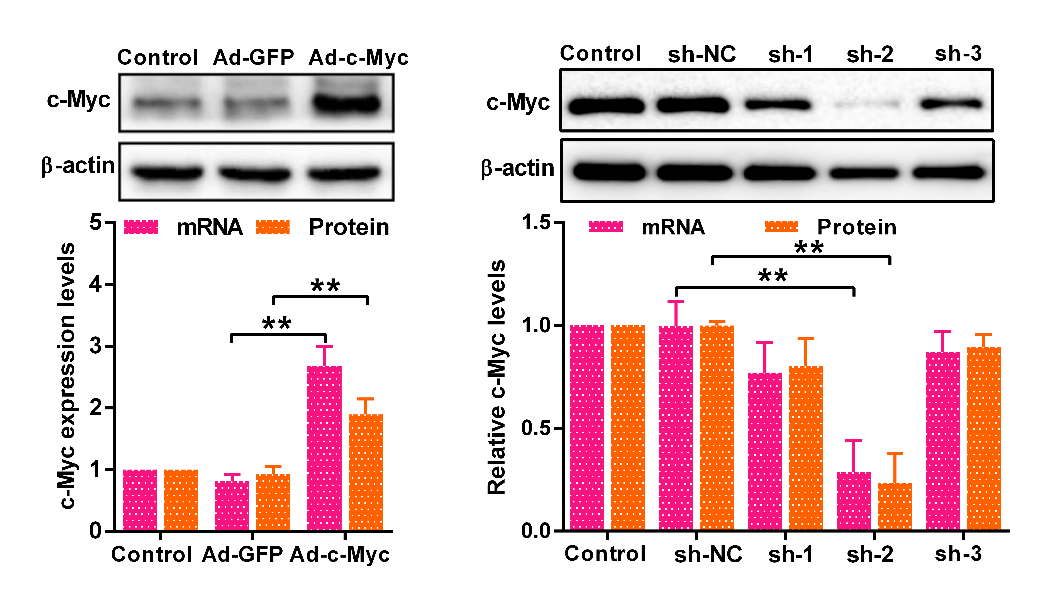


**Figure S6. c-Myc expression in aged cardiomyocytes.** qRT-PCR and Western blot analysis of c-Myc expression in aged cardiomyocytes transfected with Ad-c-Myc or sh-c-Myc (sh-c-Myc-1, sh-c-Myc-2, sh-c-Myc-3) (n=3). Data were presented as mean ± SD. ***P*<0.01.


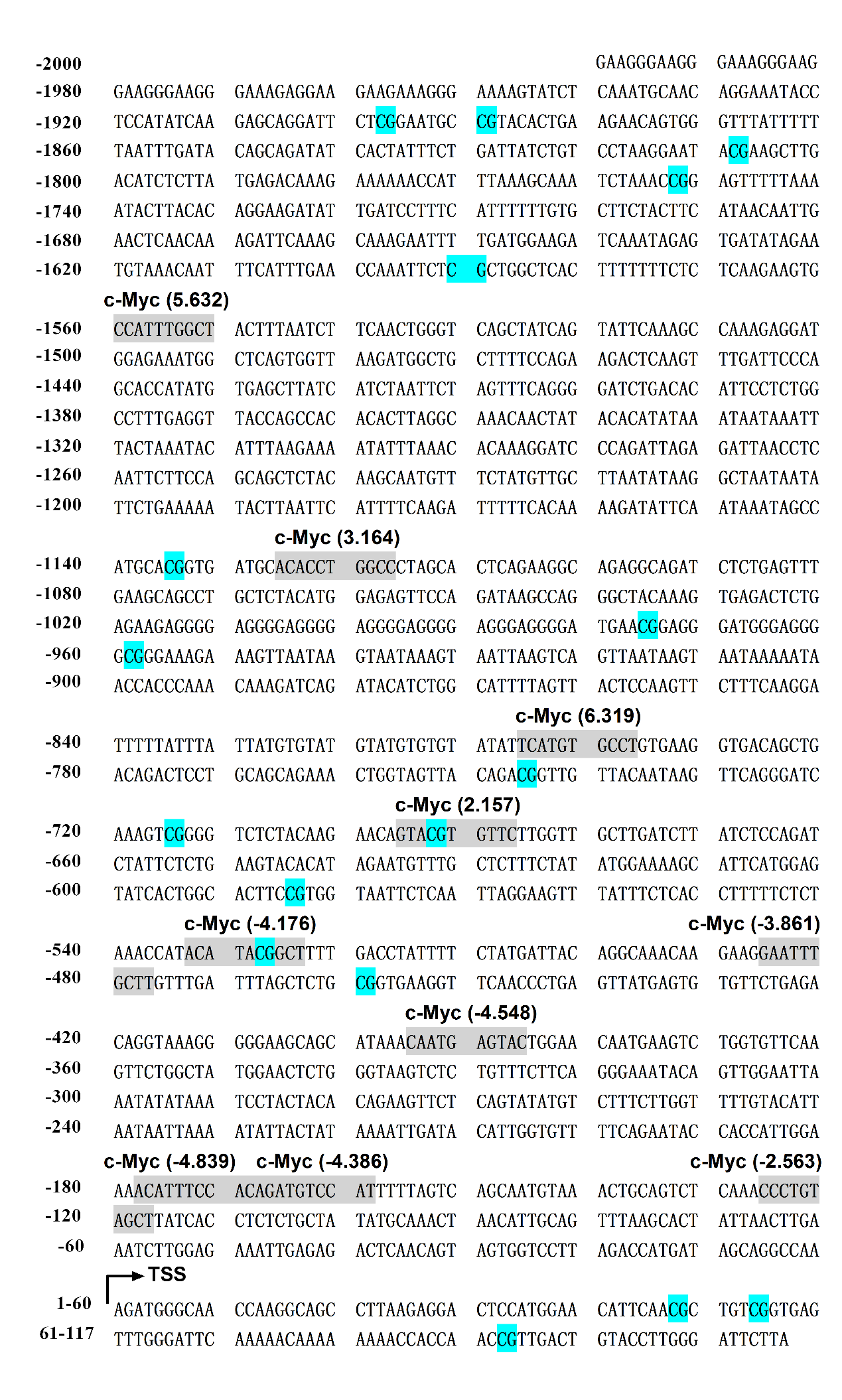


**Figure S7. c-Myc binding sites at miR-181a-2-3p promoter**. Location of the predicted c-Myc binding sites at the miR-181a-2-3p promoter region. The miR-181a-2-3p promoter (-2000/+117) was analyzed using the JASPAR database (https://ngdc.cncb.ac.cn/databasecommons/database/id/176) which gives the relative score. Ten putative binding sites of the c-Myc transcription factor are highlighted as gray, the CpG sites are highlighted as blue, and the TSS is labeled.

**Supplementary Tables**

**Table S1. Sequences of shRNAs against AMBRA1, DNMT3b,**

**HDAC2, HDAC7, HDAC11, c-Myc**

| **shRNA** | **Sequence, 5' to 3'** |
| --- | --- |
| AMBR1-1 | CCACACAUGUGAACCAUAATT |
| AMBR1-2 | CCCUAUAGAUGGAACAGAATT |
| AMBR1-3 | CCAAGCGAUUUGGUCCCAATT |
| DNMT3b-1 | GCTAGTGGGTATGAGGACTGT |
| DNMT3b-2 | GCTCTGATATTCTCATGATGC |
| HDAC2-1 | GCAAGAAGAAAGTGTGCTACT |
| HDAC2-2 | GGTATAGATGACGAGTCATAT |
| HDAC2-3 | GCCTGGTGTTCAAATGCAAGC |
| HDAC7-1 | GCGCUACAAACCCAAGAAATT |
| HDAC7-2 | CCAGCAAGAUCCUCAUUGUTT |
| HDAC7-3 | GCUGAUGGAAGAGGAAGAATT |
| HDAC11-1 | CCAGCAUGUACCAGAGAAATT |
| HDAC11-2 | GGGAUCGCUUUGCUAAAGATT |
| HDAC11-3 | GAGCUACCAUCAUUGAUCUTT |
| c-Myc-1 | GCTTCGCTAACAGGAACTATG |
| c-Myc-2 | GAATTTCTATCACCAGCAACA |
| c-Myc-3 | GGAGATGATGACCGAGCTACT |
| Negative control | TTCTCCGAACGTGTCACGT |

**Table S2. Primer sequences for qRT-PCR**

| **Gene** | **GenBank** | **Primer sequence, 5' to 3'** |
| --- | --- | --- |
| β-actin | NM_031144.3 | Forward: TGTCACCAACTGGGACGATA  Reverse: GGGGTGTTGAAGGTCTCAAA |
| AMBRA1 | NM_001134341.2 | Forward: GCTCGGAATATGGTGGCAGTG  Reverse: TGCGGTGGCTAACGATGATGTTG |
| c-Myc | NM_012603.2 | Forward: GCCTTTTCGTTGTTTTCCAA  Reverse: CACAGCAAACCTCCACACAG |
| HDAC1 | NM_001025409.1 | Forward: CGGTGCTGGACTTACGAGAC  Reverse: ATTGGAAGGGCTGATGTGAA |
| HDAC2 | NM_053447.1 | Forward: GGGCTGCTTCAACCTAACTG  Reverse: TTCACAATCAAGGGCAACTG |
| HDAC3 | NM_053448.1 | Forward: CTGGGAGGTGGTGGTTACA  Reverse: CTGGATGGAGCGTGAAATCT |
| HDAC4 | NM_053449.1 | Forward: CTCCGTGGCAAGTGTGAGT  Reverse: GTCTGTTGAGAGGGTTTGTGC |
| HDAC5 | NM_053450.1 | Forward: ATGAGGAGGAGGACGAGGA  Reverse: CTGTAACTGCTGGGCATCTG |
| HDAC6 | [XM_006256759.2](http://probes.pw.usda.gov/batchprimer3/batch_primers/58.32.217.78_1447222491/58.32.217.78_14472224916.html) | Forward: GGGAATGGAACTCAGCACAT  Reverse: CTCGCCCTACTTGGCTACTG |
| HDAC7 | XM_008765843.1 | Forward: TGTCCAGAACCCAGTCTTCC  Reverse: GTTTCAGCATCACCGAGTCA |
| HDAC8 | NM_001126373.2 | Forward: AGGAGGAGGAGGCTACAACC  Reverse: CCAGCACATAATCAGGACCA |
| HDAC9 | [NM_001200045.1](http://probes.pw.usda.gov/batchprimer3/batch_primers/58.32.217.78_1447222491/58.32.217.78_14472224919.html) | Forward: GTGGCAGAGAGGAGAAGCAG  Reverse: TGTTTGGTGAACTGGGACCT |
| HDAC10 | NM_001035000.1 | Forward: TTCCAGCCTGACATGGTGTTGATG  Reverse: TCCGAAGCATTGCAGCCAAGAG |
| HDAC11 | NM_001106610.2 | Forward: GGCACGAGCGAGACTTCATGG  Reverse: CAACTCCACCTTCCGCCTGATG |

**Table S3. Primer sequences for nMS-PCR**

| **Primer set** | **Primer sequence, 5' to 3'** |
| --- | --- |
| miR-181a-2-3p-O | Forward: TTTTGTAGTAGAAATTGGTAGTTATAGA  Reverse: AATCATAAAAAATAAATCAAAAACC |
| miR-181a-2-3p-M | Forward: TATGGAGTATTATTGGTATTTTCGT  Reverse: ATAACTCAAAATTAAACCTTCACCG |
| miR-181a-2-3p-U | Forward: TATGGAGTATTATTGGTATTTTTGT  Reverse: TAACTCAAAATTAAACCTTCACCAC |

O, out primer; M, methylation primer; U, unmethylation primer.

**Table S4. Primer sequences for ChIP assay**

| **Genes** | **primer sequence, 5' to 3'** |
| --- | --- |
| miR-181a-2-3p  (-1126/-1117) | Forward: ACCTCAATTCTTCCAGCAGCTC  Reverse: TCTGCCTCTGCCTTCTGAGTG |
| miR-181a-2-3p  (-806/-797) | Forward: CCCAAACAAAGATCAGATACATCTG  Reverse: AACCGTCTGTAACTACCAGTTTCTG |
| miR-181a-2-3p  (-774/-765) | Forward: CAATAAGTTCAGGGATCAAAGTCG  Reverse: CACGGAAGTGCCAGTGATACTC |
